# Assessing the implementation of a multi-component hypertension program in a Guatemalan under-resourced dynamic context: An application of the RE-AIM/PRISM extension for sustainability and health equity

**DOI:** 10.21203/rs.3.rs-2362741/v1

**Published:** 2023-01-17

**Authors:** Alejandra Paniagua-Avila, Rachel C. Shelton, Ana Lissette Guzman, Laura Gutierrez, Diego Hernandez Galdamez, Juan Manuel Ramirez, Javier Rodriguez, Vilma Irazola, Manuel Ramirez-Zea, Meredith P. Fort

**Affiliations:** Columbia University Medical Center: Columbia University Irving Medical Center; Columbia University Mailman School of Public Health; INCAP: Instituto de Nutricion de Centroamerica y Panama; IECS: Instituto de Efectividad Clinica y Sanitaria; INCAP: Instituto de Nutricion de Centroamerica y Panama; INCAP: Instituto de Nutricion de Centroamerica y Panama; INCAP: Instituto de Nutricion de Centroamerica y Panama; IECS: Instituto de Efectividad Clinica y Sanitaria; INCAP: Instituto de Nutricion de Centroamerica y Panama; CU Anschutz Medical Campus: University of Colorado Anschutz Medical Campus

**Keywords:** Implementation science, hypertension, Latin America, low- and middle-income countries, mixed methods, RE-AIM/PRISM, health equity, sustainability, dynamic context, adaptations, hybrid trial

## Abstract

**Background:**

The COVID-19 pandemic necessitated rapid changes in the delivery of care across public primary care settings in rural Guatemala in 2020. In response, a hypertension program implemented within the public primary care system required multiple adaptations, providing an illustrative example of dynamic implementation amidst changing context in an under-resourced setting. This study describes the evolvability of an evidence-based intervention (EBI; protocol-based hypertension treatment) and one of its main implementation strategies (team-based collaborative care) during the COVID-19 pandemic and discusses implications for health equity and sustainability.

**Methods:**

This convergent mixed methods analysis assessed implementation across five Ministry of Health districts during the initial phase of the pandemic. Qualitative and quantitative data were collected, analyzed, and integrated, informed by the RE-AIM (Reach, Effectiveness, Adoption, Implementation Maintenance) Framework’s extension for sustainability, and its contextual enhancement, PRISM (Pragmatic, Robust, Implementation and Sustainability Model). For RE-AIM, we focused on the “Implementation” domain, operationalizing it qualitatively as continued delivery and adaptations to the EBI and implementation strategy, and quantitatively as the extent of delivery over time. We conducted 18 in-depth interviews with health providers / administrators (n=8) and study staff (n=10) and performed a matrix-based thematic-analysis. Qualitative results informed the selection of quantitative implementation summarized as behavior over time graphs. Quantitative implementation data and illustrative quotes are presented as joint displays.

**Results:**

In relation to implementation, several organic adaptations hindered delivery, threatened sustainability, and may have exacerbated health inequities. Planned adaptations enhanced program delivery and may have supported improved equity and sustainability. Salient PRISM factors that influenced implementation included “Organizational perspective of the EBI”, “Fit” and “Implementation and sustainability infrastructure”. Facilitators to continued delivery included the perception that the EBI is beneficial, program champions, and healthcare team organization. Barriers included the perception that the EBI is complicated, competition with other primary care activities, and temporary suspension of services due to COVID-19.

**Conclusions:**

Multi-level contextual changes led to numerous adaptations of the EBI and implementation strategy. Systems thinking approaches may shed light on how a program’s sustainability and its equitable delivery are influenced by adaptations over time in response to dynamic, multi-level contextual factors.

**Trial registration:**

NCT03504124

## Background

To shed light on the nature of successful implementation efforts, implementation researchers have called for an improved understanding of the interplay between interventions, implementation strategies and multi-level context (1). Implementation scientists have posited that interventions are not discrete packable units that may be implemented in any setting, regardless of context (2). The role of evolving, multi-level context in implementation efforts is increasingly recognized, and has been discussed in detail in frameworks like the Dynamic Sustainability Framework (2). Such frameworks question the idea that interventions can and should be fully optimized during the pre-implementation phase and then implemented with full fidelity (2). Instead, such an approach recognizes the value of learning from and adapting to dynamic contexts and evidence over time, and the potential importance of the evolvability of interventions and implementation strategies in response to changing contexts (2–4).

Along these lines, sustainability, the extent to which an intervention continues to be delivered and continuously delivers its intended benefits over an extended period of time, is an essential focus of implementation science (2,3). Within this scenario, the COVID-19 pandemic emerged, requiring drastic and rapid changes to healthcare settings and communities at large, overburdening already strained systems and healthcare workers. The COVID-19 pandemic threatened the sustainability of evidence-based interventions (EBIs) for chronic health conditions beyond COVID-19 and highlighted pre-existing health and social inequities (5,6). To implementation scientists, the COVID-19 pandemic provided a striking example of a crisis that provoked significant changes to dynamic contexts and an opportunity to better understand how contextual changes influence EBIs and implementation strategies. It is particularly urgent to study how EBIs, implementation strategies and dynamic contexts interplay and may be evaluated in low- and middle-income countries (LMICs), where public health and primary care systems are disproportionally under-resourced.

Since 2018, our team has been studying the effectiveness and implementation of a multi-component hypertension program delivered within the public primary care system in rural Guatemala through a hybrid type 2 cluster randomized control trial (cRCT) (7). This effort is being led by the Institute of Nutrition of Central America and Panama (INCAP) in partnership with Guatemala’s Ministry of Health and Social Welfare (MoH) (8). The multilevel multicomponent hypertension intervention program (thereafter referred to as “program”) consists of a core evidence-based intervention (EBI), protocol-based stepped-care hypertension treatment, and five implementation strategies, which have been previously described (7,9,10). In March 2020, 9 months after launching the program, COVID-19 cases were growing in Guatemala and a national-level response with lockdowns and significant disruptions to rural communities and healthcare services occurred. As in other LMICs, Guatemalan public primary care services were disrupted and necessitated rapid and drastic changes, providing a remarkable example of a rapidly evolving context. During this period, we conducted our first planned implementation assessment of the program using the contextually-expanded RE-AIM/PRISM framework (11). As COVID-19 enhanced existing health inequities and threatened the sustainability of programs for chronic diseases like hypertension, we conducted this assessment following the recent extension of RE-AIM by Shelton and colleagues (2020) (4), which provides explicit guidance in bringing a focus on sustainability and health equity to implementation indicators and metrics. The present manuscript presents results from this implementation assessment, guided by RE-AIM/PRISM and sustainability and health equity perspectives, conducted within five of the 18 municipal health districts that were implementing the program within the rural public primary care system in Guatemala.

## Methods

### Design

We used a convergent mixed methods design to assess implementation of the multicomponent hypertension program during the initial months of COVID-19 (12). Data collection focused on program implementation at the healthcare facility level from the providers and study staff perspectives. Table 1 summarizes the frameworks, selected dimensions, questions and data sources evaluated in this study.

This study was approved by the Guatemalan National Ethics Committee, INCAP Institutional Review Board (IRB), Columbia University Irving Medical Campus IRB, and the University of Colorado IRB.”

### Implementation science frameworks

As shown in table 1, we utilized the RE-AIM extension for sustainability and PRISM framework to guide this assessment (4,11). RE-AIM includes five implementation dimensions – reach, effectiveness, adoption, implementation and maintenance – at the participant and implementer levels, that are critical to producing more widespread implementation and broader population health impact (4,13,14). In this assessment, we focused on RE-AIM’s “implementation” dimension, operationalized as program delivery + program adaptations (15). The RE-AIM extension on sustainability was pertinent given that the COVID-19 emergency was leading to rapid changes to the context where the program was being implemented, providing an opportunity to better understand the implications for health equity and sustainability. PRISM, a contextual expansion of RE-AIM, was also used here to guide understanding of the multi-level factors that influence RE-AIM outcomes such as fit between the intervention and context, and the implementation and sustainability infrastructure (11,16).

### Study Setting

**Figure 1:**
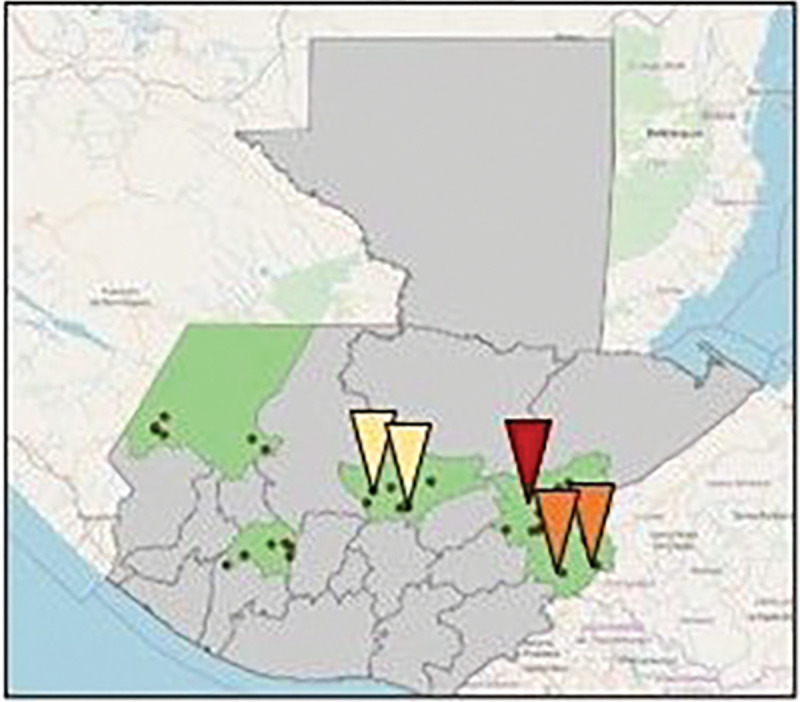
Study setting. Five health districts implementing the hypertension program that are included in this analysis are identified with yellow, red and orange arrows. These districts are in 3 of the 5 Ministry of Health areas (departments) in the parent study that are shaded green.

We conducted this study in five of the 18 intervention health districts (clusters) in the parent study. Guided by field study staff, we selected five health districts in the three Health Areas with the longest experience implementing the program representing a wide range of program implementation (e.g. low and high implementers) Figure 1 shows the parent study’s five departments (that correspond to Ministry of Health Areas) shaded in green and the red, orange and yellow arrows show the five Ministry of Health districts selected for this analysis. Each health district roughly corresponds to a municipal-level geographic area,

### Participants

To comprehensively assess implementation from different perspectives and levels, we conducted interviews with people who were closely involved in program implementation at the health area administration (department- or provincial-level), municipal health districts (municipal-level) and health posts (community-level). We also included Ministry of Health (MoH) staff responsible for health management and service provision, and INCAP study staff responsible for supporting program implementation and gathering data. Study staff were invited to participate by APA, while MoH staff were invited to participate in the study by research assistants. All participants provided verbal informed consent prior to initiating the qualitative interviews.

### Data collection

#### Qualitative data collection

As shown in the Study summary box, the qualitative in-depth interviews were structured around the RE-AIM extension for sustainability and PRISM frameworks. We started by asking participants to describe the hypertension program in their own words and to share their perspectives about it. Later, we asked about the implementation of the EBI / implementation strategies before and during COVID-19, focusing on the adaptations and reasons behind such adaptations, with implications for both implementation and future sustainability. To assess fit, we inquired about the organizational factors that may have influenced program implementation, guided by PRISM. To assess the implementation and sustainability infrastructure, we asked participants to describe what needs to be in place for the program to be implemented and sustained during and beyond the study period. We asked about potential sources of inequitable program implementation, and ways to overcome them. Finally, participants shared lessons learned and recommendations to sustain the program beyond the study period.

**Figure 2:**
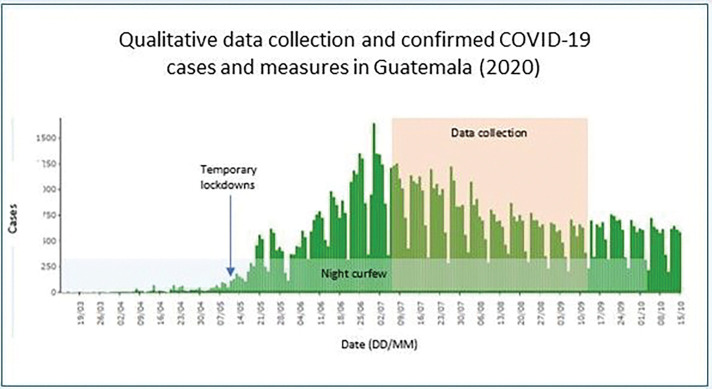
Timing of qualitative data collection in relation to confirmed COVID-19 cases and COVID-19 control measures in Guatemala (2020)

Interviews were conducted by APA and ALG in Spanish; audio recorded over the phone; between July and September 2020; 9–12 months after the program was launched; and 3–5 months after the start of the lockdowns due to the COVID-19 epidemic Figure 2 shows the timing of qualitative data collection in relation to confirmed COVID-19 cases and COVID-19 control measures in Guatemala from mid-March to mid-October 2020.

#### Quantitative data collection

We collected quantitative data corresponding to key indicators of program implementation. Specifically, we focused on program delivery of the EBI over time at the healthcare facility level (health post and health center), measured as the proportion of observed vs expected number of hypertensive medications per time period. We also assessed the delivery of the implementation strategy over time at the healthcare facility level, measured as the percentage of the observed vs the expected number of team meetings per time period. MoH providers in charge of delivering the program collected quantitative data on paper-based forms that were previously co-designed and piloted by the research team and MoH staff. Every three months, study staff reviewed and entered the paper-based data into a ReDCap electronic database hosted by INCAP (17,18).

### Data analysis

#### Qualitative data analysis

To analyze qualitative data, we followed the Rapid Identification of Themes from Audio Recording (RITA) methodology and the matrix-based approach to thematic-analysis (19,20). RITA consists of identifying pre-defined themes from audio recordings without the need to have verbatim interview transcriptions and line-by-line coding (19). In our study, RE-AIM / PRISM domains informed the development of deductive categories, while leaving space for unanticipated inductive findings (Table 1). Following RITA, analysts (APA and ALG) independently listened to interview recordings taking notes in interview templates that were reviewed and consolidated into one summary per interview by ALG. Later, analysts transferred information from interview summaries to a matrix that consolidated all the information for health district. Analysis was conducted by a multidisciplinary team with expertise in qualitative methods (all), medicine and public health (APA), chronic diseases (ALG), health systems (MPF) and implementation science (MPF). This team met weekly to discuss results and implications for sustainability and health equity. Other co-authors were consulted at key points in the analysis to share their expertise and insight.

#### Quantitative data analysis

Our quantitative data analysis focused on the RE-AIM “implementation” domain, operationalized as the extent of program (EBI and implementation strategy) delivery over time. We analyzed facility-level indicators of program delivery to shed light on important metrics of program implementation over time and across districts. After selecting the indicators, we built behavior-over time graphs to explore the level of program delivery across districts and over time, particularly before and during-COVID-19 (21).

### Qualitative and quantitative data convergence

Finally, we converged qualitative and quantitative results to see if they reached the same conclusion (12,22) . We selected illustrative quotes, themes and quantitative summary data in joint displays focusing on the implementation of the core program component (protocol-based hypertension treatment) and one of the implementation strategies (team-based collaborative care) (23).

## Results

Table 1 summarizes the key informants who were interviewed for this assessment. We conducted 18 in-depth interviews with MoH staff and INCAP study staff. Interviewees represented five health districts and were involved in the program implementation from different levels of the health system, including the community-, health facility- to the health administration levels.

Table 2 presents a summary of the key qualitative results organized by the PRISM and RE-AIM dimensions that we focused on for this analysis: organizational (health district) perspectives on the hypertension program, fit between the EBI and the health district, implementation and sustainability infrastructure, and implementation (with an emphasis on adaptations).

**Figure 3:**
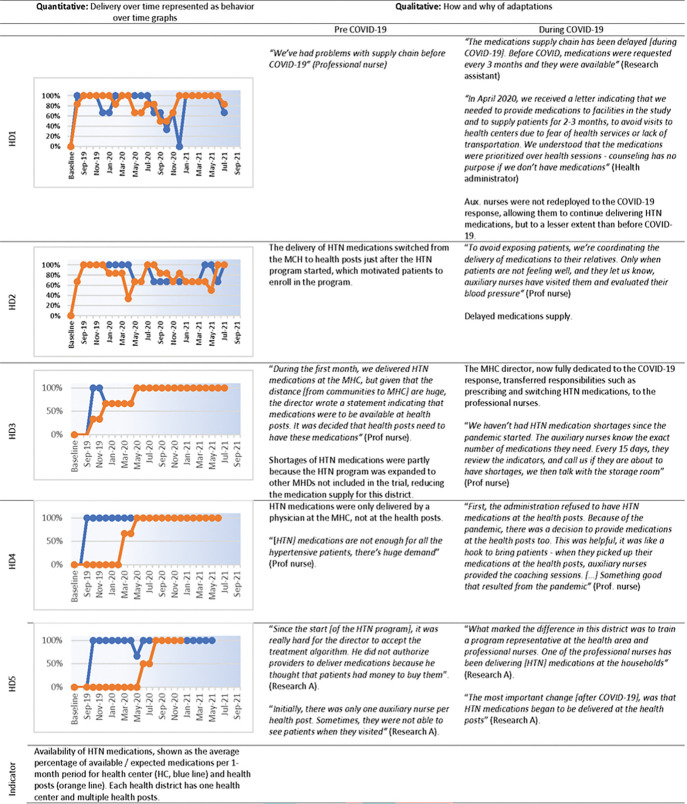
Availability of medications for protocol-based hypertension treatment (evidence-based intervention) at each municipal health district (HD1 to HD5), before (light background) and during COVID-19 (dark background). HTN: Hypertension; HD: health district; MoH: Ministry of Health; Research A: Research Assistant; Prof nurse: Professional nurse

**Figure 4:**
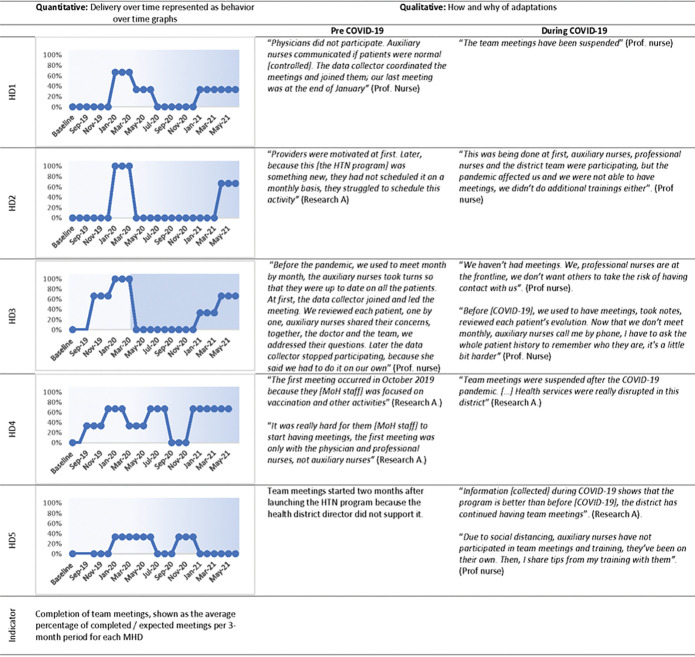
Team meetings for team-based collaborative care (implementation strategy), at each health district (HD) before and during COVID-19 (shadowed background). HTN: Hypertension; HD: health district; MoH: Ministry of Health; Research A: Research Assistant; Prof nurse: Professional nurse

Figures 3 and 4 are joint displays that present quantitative and qualitative results related to the implementation domain. They are described in detail in the text below. Behavior-over-time graphs summarize quantitative results on the delivery of the EBI or implementation strategy over time with blue lines representing the district-level health center and orange lines representing a health post within the district. Adjacent columns include select quotes describing adaptations to the EBI or implementation strategy before and during COVID-19.

### Implementation

#### Implementation of protocol-based hypertension treatment (EBI)

Implementation of the protocol-based hypertension treatment (EBI) evolved, before and during-COVID-19, as indicated by the availability of hypertensive medications at healthcare facilities (Figure 3). BOT graphs show that the availability of hypertensive medications increased shortly after launching the program at all of the health districts included in this assessment. Later, availability differed by health district. Qualitative data about the how and why of adaptations provided potential explanations for differences in BOT graphs. Taking health district 2 (HD2) as an example, before COVID19, administrators worked to ensure the availability of hypertensive medications at the health center and health posts to facilitate patient access, an example of a planned adaptation needed to implement the program over time. Later, during COVID19, supply chain disruptions reduced the availability of hypertensive medications, an example of an organic contextual adaptation that occurred and impacted implementation. In contrast, health district 3 (HD3) had sufficient hypertensive medications prior to and during COVID19 due to frequent medication surveillance and coordination between health centers and health posts to sustain the medication supply chain, an example of a planned adaptation in the context of COVID-19.

#### Implementation of team-based collaborative care (implementation strategy)

The implementation of team-based collaborative care evolved over time as indicated by the completion of team meetings. Figure 4 shows quantitative and qualitative differences between pre- and during COVID19 periods. BOT graphs show that in health districts 1–3, the implementation of team meetings was greater than 60% at least once before COVID19 but dropped to 0% during COVID19. In contrast, health districts 4–5 had lower implementation of team meetings before and during COVID19. Qualitative data confirmed and expanded on the quantitative results. For instance, team meetings were suspended during COVID19 to keep social distance (HD3, planned adaptation) or because healthcare services were suspended (HD4, organic contextual adaptation). In contrast, in health district 5 (HD5), the delivery of team meetings was lower than at other health districts at all times because leadership did not support the program. Qualitative data also provide details on adaptations to the implementation strategy, complementing the quantitative information. For example, during COVID19, the BOT graph for HD3 shows that the meetings dropped to 0%. Qualitative data, however, show that despite the suspension of team meetings, this health district continued carrying out the function of the team-based collaborative care strategy, as healthcare providers continued coordinating with each other by phone in order to provide team-based collaborative care, an example of a planned adaptation in the context of COVID19.

### Organizational perspectives of the hypertension program

We asked participants to describe the program and share their perceptions about it. We found that the perspectives of healthcare providers / administrators (i.e. the implementers) appeared to influence program implementation (delivery and adaptations) and the intention to sustain it. Perspectives varied widely between health districts and type of healthcare provider / administrator. As mentioned above, in HD5, the lack of support from leadership delayed the launching of team-based collaborative care meetings ( implementation strategy) and other program elements (See figure 4). In a different district (HD1), an observed facilitator was that healthcare administrators and nurses perceived it as “beneficial to community members” and a “learning opportunity”. Whereas in that same district, a barrier was that physicians in HD1 perceived the program to be an “additional workload” and were minimally involved in program delivery. Thus, program implementation was adapted to the context, as professional nurses took on physicians’ responsibilities, such as evaluating patients, prescribing medications and coordinating team-based collaborative care. These adaptations allowed the program to be implemented in this district, overcoming the lack of support from physicians.

“Physicians did not buy into it, they did not participate in the program and some staff perceived the program as “additional workload”. Professional nurses ended up taking on responsibilities that had been assigned to physicians.”

– Data collector, HD1

If healthcare providers and administrators perceived the program as beneficial and feasible, they appeared to support its institutionalization (with implications for sustainment) within the MoH beyond the study period.

“I think that the MoH can continue [implementing the program], as can help patients and prevent [hypertension] - patients’ relatives are trying to understand how to improve their health without medications, they want to start taking action. Patients go back home and share their experiences with their relatives on how to prevent chronic diseases – diet, salt consumption, weight, exercise, many habits that should be avoided, like eating junk food. This is very important and should be sustained. The MoH should take it on as its own program”

– Health district administrator, HD3

### Fit between hypertension program and health districts

We asked participants to explain how the characteristics of health districts influenced program implementation (fit), which we categorized into barriers and facilitators related to fit. A major barrier related to fit was competition with other MoH primary care program activities. Two other barriers that we observed were: insufficient and overburdened healthcare staff and the temporary suspension of healthcare services during the initial part of the COVID-19 pandemic. Facilitators related to fit were: previous experience providing services for chronic diseases, the presence of program champions and strong leadership, and the extent of organization, collaboration and communication within the district team. At the time that the interviews were conducted, the number of COVID19 infections were on the rise and providers were increasingly becoming involved in the pandemic response, which presented an overarching challenge for program fit across health districts.

#### Competition with other MoH primary care programs

Across all health districts, MoH providers and administrators described the difficulties of being responsible for delivering 22 other MoH programs at the primary care level, in addition to the hypertension control program. Providers described the tensions between delivering care for acute problems and maternal and child programs as compared to care for chronic conditions, such as hypertension. Historically, maternal and child health programs have been prioritized over chronic diseases. Often, providers had to choose between delivering one program over the other one. For example, a data collector at HD2 described that providers had to miss meetings for the team-based collaborative care in order to respond to acute health problems (e.g. an undernourished child), reducing the delivery of this implementation strategy. Moreover, the COVID19 pandemic response initially led to the suspension of healthcare services for chronic diseases, and later created more responsibilities among the primary care team, reducing program fit for the district.

*“During the initial phase [of COVID-19], services for patients with chronic diseases were suspended, all at once we had 13 COVID cases, we were afraid.”* -Professional nurse, HD3

*“Remember that an auxiliary nurse is responsible for 22 other MoH programs, and now, with this pandemic it’s even worse.”* -Professional nurse, health area administration for HD1, 2

#### Previous experience providing services for chronic diseases

The program fit was facilitated by the particular health district’s experience providing care for chronic cardiometabolic diseases (e.g. diabetes mellitus 2 and hypertension). MoH administrators responsible for overseeing multiple districts, highlighted that districts with previously existing chronic disease programs had capacities and facilitators to implement the hypertension program. For instance, the healthcare team was already trained in chronic diseases, accustomed to and organized in such a way that allowed them to follow-up with chronically ill patients. In contrast, other healthcare teams were only organized to provide care for acute health problems (see previous theme). Providers within districts with previous experience providing services for chronic diseases (HDs 1–3) agreed with the administrators and indicated that the hypertension program was an improvement over their previous program, rather than a completely new approach to treating hypertension.

*“At HD1, this isn’t a new program. Since 2011 we’ve been following-up with patients with chronic diseases, monthly or annually, as part of the Inclusive Health Model* (Modelo Incluyente de Salud, MIS), *which is supported by the Institute for Inclusive Health. […] Implementing this program has not been very hard for our district. This new program improved our approach to managing hypertension.”* – Professional nurse, HD1 administrator

#### Program champions and strong leadership

Most participants (MoH and study staff) highlighted that strong leadership and program champions enhanced the program fit to the health districts. Program champions were MoH leaders, such as health area or health district administrators, who strongly supported the hypertension program and mobilized their teams to implement it. They also actively adapted the EBI / implementation strategy and ensured that key infrastructure elements were available, which enhanced the program’s fit to their health district (see implementation and sustainability infrastructure below). For example, the medical director of HD3 ensured the availability of hypertensive medications at health centers and health posts, facilitating the implementation and fit of the protocol-based hypertension treatment (See Figure 3). Program champions also facilitates the program’s expansion and sustainability potential. For example, the medical director at HD3 promoted the program expansion to additional health facilities, adapted it to manage diabetes mellitus 2 in addition to hypertension, and communicated the importance of sustaining the program beyond the study period to healthcare providers.

*“The health area administrator [in charge of HD 1, 2] really supports the program and she has made sure that hypertensive medications are available, which motivates patients to participate in the program.”* - Data collector, HD2

*“Other health district directors would say ‘Let’s only implement this program for a short time’, but instead she [referring to the HD medical director] motivates us, and we motivate the auxiliary nurses to deliver the hypertension program. […] Professional nurses trained by the study team trained other professional nurses responsible for other health posts. Now the hypertension program is being implemented within the entire health district.”* – Professional nurse, HD3

### Implementation and sustainability infrastructure

We asked participants to reflect on what would be needed for the program to be implemented and sustained over time. We identified two primary components of the implementation and sustainability infrastructure, which are described below: human resources, and equipment and medications. These and other aspects of the implementation and sustainability infrastructure are presented as resources and processes in Table 2.

#### Human resources

The need for additional human resources to deliver the program was a recurring theme in all the health districts. Participants identified the need for additional providers to deliver care for chronic diseases like hypertension, in addition to the rest of the MoH primary care programs (e.g. childhood immunizations). Some participants suggested that health districts would need at least three auxiliary nurses per health post, instead of one or two. Moreover, to sustain the program beyond the study period, participants suggested creating the role of a local-level chronic disease program coordinator, who would be in charge of helping patients navigate care by scheduling their health coaching sessions, monitoring the availability of hypertension medication at health facilities, and coordinating between providers based at health centers (e.g. physicians) and those based at the health posts (e.g. auxiliary nurses). Providing ongoing training, supervision and support to auxiliary nurses was also identified as a need.

*“Our main challenge to deliver this program is that we need more human resources.”* – Health area nurse, HD1, 2

*“The program can be implemented as planned, but there needs to be a supervisory team from the MoH in charge of monitoring the program, supervising and training [healthcare providers].”* – Research assistant, HD3

#### Essential equipment and hypertensive medications

Participants identified the need for equipment (e.g. blood pressure monitors) and recognized that prior to the start of the program such equipment was not available or working at most health facilities. Most participants highlighted that ensuring the availability of hypertensive medications had been challenging during program implementation, and particularly during COVID19. In order to sustain the program beyond the study period, hypertensive medications would need to be available, which would require health districts and health areas to improve their medication supply chain.

*“This program has been possible just because INCAP provided us with blood pressure monitors, weight balances…”* – Health area nurse, HD 4, 5

### Health equity considerations related to the RE-AIM implementation domain

To identify instances of inequitable program implementation and reach, we asked participants to describe differences in program implementation between facilities, communities and individuals. Multiple participants provided examples of differences in program implementation between and within health districts. Those health districts or health facilities which approved the program and had sufficient infrastructure (e.g. human resources) seemed to deliver the program more successfully than their counterparts. Participants identified individual and community characteristics that may have led to inequitable implementation and reach of the program, including poverty, rurality (mountainous areas, larger distance to healthcare facilities) predominantly Maya-speaking populations, and in communities that had lack of support from community leaders. Participants described individual-level characteristics that may have led to inequitable participation, including working in the agricultural sector, lacking formal education, lacking family support to engage in program activities, and being a female with an authoritative husband.

*“It is easier with patients who have a relative who is willing to help them, if they know how to read and write, and if they live closer to the health services. Patients without family support may not receive hypertensive medications”* -Data collector, HD2

*“Some female participants required husband authorization to enroll in the program. Some were not authorized and did not enroll in the program”* – Data collector, HD1

### Sustainability considerations related to the RE-AIM implementation domain

We asked participants to reflect on the program’s sustainability beyond the study period, considering the context of COVID-19. While participants identified threats to program sustainability, most of them stated that it was feasible to sustain the program. Health administrators showed interest and commitment to continuing implementation of the program beyond the study period and health providers recognized the importance of doing so. As described above, participants identified the need to meet with essential infrastructure elements for the program to be sustained. Participants also reiterated the need to recognize and integrate the hypertension program within the MoH primary care programs. In addition, for the program to be sustainable, health districts would need to ensure transportation for providers to visit patients in the most mountainous and rural areas. Finally, to continue implementing the program in the context of COVID19, it would need to undergo planned adaptations to improve its feasibility and reach, such as delivering health coaching sessions in a group setting, rather than individually.

*“The program should be expanded beyond the patients who are enrolled, for everyone - this is really important. This is a pilot, which allows us to evaluate the results and make recommendations to the MoH so that they know what to do. But we need to go beyond recommendations, we also need to deliver training workshops, buy equipment and supplies, hire staff designated to this [program]”* –Professional nurse, HD4

*“It is possible to continue delivering the program within the new COVID19 reality. First of all, we need HTN medications. Second, we need training to provide health coaching sessions – we already have that, and we need to strengthen it. Third, we need to continue delivering health coaching sessions, but now as part of patient clubs.”* – Health area nurse, HD3

## Discussion

Our mixed methods analysis of mid-course implementation and context of a new hypertension control program within Guatemala’s public primary system during the initial months of the COVID-19 pandemic led to two major conclusions. First, we documented a close interplay between the rapidly evolving context, which influenced the extent of delivery and adaptations to the EBI and the implementation strategy. Second, we identified sources of unequal program implementation and potential threats to program sustainability as well as opportunities for improving health equity and sustainability.

We observed the close interplay between contextual factors, EBIs and implementation strategies pointed out by others (1). We found that contextual factors influenced program implementation (delivery and planned/organic adaptations). For instance, altering the implementation and sustainability infrastructure by providing basic program equipment (e.g. blood pressure monitors) and hypertensive medications prior to launching the program, facilitated an initial increase in program delivery. In contrast, reduced availability of hypertensive medications during COVID-19, led to reduced program delivery. In addition to influencing program delivery, contextual changes led to program adaptations. To respond to the new COVID-19 context, healthcare providers implemented program adaptations, some of which seemed to increase the fit between context and program. For example, to overcome challenges due to transportation disruptions and social distancing, healthcare providers began delivering hypertensive medications at the village level, bringing them closer to patients. Our study showed that rapid contextual changes at the community and primary care levels led to drastic changes in program delivery, which spurred planned program adaptations. In turn, such adaptations allowed the program to be delivered within the new context. In future studies, utilizing system dynamics approaches could help to understand feedback loops and dependencies between contextual factors, EBIs, and implementation strategies, as well as the points that may be leveraged to improve implementation and clinical outcomes.

Similar to other implementation assessments conducted during COVID-19, we found that the COVID-19 emergency further stretched an overburdened and under-resourced primary care system (8, 9), threatening continued program implementation and its future sustainability. However, even though resources and time allocated to the program decreased during COVID-19, certain health districts were able to bounce back to increase program delivery close to pre-COVID-19 levels. Understanding the factors that increased program delivery during the implementation phase, may help to draw sustainability implications. For example, we found that health districts with certain contextual factors seemed to be able to revamp the program delivery during COVID-19. Such factors included the MoH staff’s perception of the program as beneficial; essential infrastructure, such as sufficient human resources; and a strong leadership and experience implementing programs for chronic diseases like hypertension. Certain adaptations that helped with program delivery during the implementation phase may be prioritized during the sustainability phase. For instance, we learned that the implementation strategy (team-based collaborative care) may be adapted by having primary care teams (e.g. physicians and auxiliary nurses) communicate by phone multiple times a week instead of holding monthly in-person meetings. This adaptation could allow teams to alter the “form” of this strategy as needed, while still meeting with its function of making team-based decisions regarding hypertension management. In line with a dynamic understanding of sustainability, this study suggests that assessing a program under different scenarios during its implementation phase (e.g. before and during COVID-19) may provide insights for program sustainability, such as the contextual factors and program adaptations or refinements that may be needed to sustain it.

Following calls to utilize a health equity lens in implementation assessments (24), this study found that program implementation was influenced by social and structural determinants of health, such as poverty, gender, rurality and historically discriminated ethnic groups. While our program was designed and implemented with socially disadvantaged groups in mind (rural and Maya-indigenous populations served by the public primary care system), our results suggested that program adaptations may be needed to address health disparities within these groups. Importantly, certain program adaptations may lead to a more equitable program implementation, particularly the ones that allow for flexible implementation of program components to adapt to the unique needs of socially disadvantaged groups. For example, diversifying the ways of delivering hypertensive medications (e.g. through relatives, at home, at the health post) may be a way to address challenges to medication delivery. Our explicit focus on health equity in this implementation assessment, surfaced sources of health disparities and potential ways to address them through program adaptations. However, historical and broader sources of health disparities (e.g. ethnic discrimination) will not be addressed through one specific program and would require system-level or broader policy changes.

Our study has several strengths. First, we assessed program implementation over time focusing on two different phases (before and during COVID-19), which allowed us to understand how rapidly changing contextual factors led to both the EBI and the implementation strategy’s evolution. Second, we applied widely recognized implementation frameworks, RE-AIM/PRISM, coupled with an explicit focus on health equity and sustainability. Third, we utilized a mixed methods approach, interviewed participants at different levels of the health system (e.g. providers, administrators, program evaluators), and included a range of health districts, all of which allowed for a deeper understanding of the program’s implementation within the public primary care system in Guatemala. However, our results need to be interpreted in light of certain limitations. First, our analysis only included five of the 18 districts that implemented the program. However, we purposefully selected those with the most implementation experience and those representing high and low implementers. It is important to note that the rest of the health districts included in the study and districts across Guatemala have many more distinct characteristics that could change their implementation experience. Additionally, this particular assessment did not include perspectives from program recipients (i.e. patients), although our study team has captured patient perspectives in a different analysis (25).

## Conclusions

This study contributes to calls to advance our understandings of sustainability and health equity in implementation science and provides a rich empirical example of application of complementary implementation science theories and frameworks. Our study also provides urgently needed information on how to assess multi-component programs in settings with limited resources and under rapidly changing contexts, such as the COVID-19 pandemic. We conducted a rich mixed methods assessment of a program implementation over time, showing the close inter-relationships between context, EBI and implementation strategy, and their influences on health equity and sustainability. These findings point to the need for robust mixed methods and more rapid assessments, and systems science approaches that help understand the dynamic relationships between contextual and implementation factors over time.

## Figures and Tables

**Table 1.** Key informant interviews

Type of key informant	Number of interviews

Ministry of Health (MoH) staff	

Ministry of health advisor	1
Health area administrator	3
Municipal health district coordinator / provider	4

INCAP study staff	

Research coordinator	5
Data collector	5

Total	18

**Table 2.** Summary of qualitative findings by PRISM and RE-AIM dimensions

Dimension	Overview of themes and sub-themes	

**PRISM**		

Health district’s (HD) perspective on the hypertension (HTN) program	Facilitators • Perceiving the HTN program as effective or beneficial to community members engages HCPs in the program implementation.	Barriers • Perceiving the HTN program as additional workload, an imposed activity, or complicated, hinders HCP’s engagement in the program implementation and lack of support from leadership in some districts.

Fit between HTN program and HD	Facilitators • Previous experience providing services for chronic diseases in the HD • Program champions and strong leadership at the MHD-level and health area level • Healthcare team organization, collaboration and communication	Barriers • Competition between HTN program and other MoH primary care programs • Insufficient and overburdened healthcare staff • Temporary suspension of healthcare services due to COVID-19
	
		

Implementation and sustainability infrastructure	Resources • Essential equipment and supplies to deliver the program (e.g. blood pressure monitor) • Essential human resources to deliver the program and 22 other primary care programs • Transportation allows providers to visit patients not able to visit the health post • Financial resources to cover programs for chronic diseases, including hypertension	Processes • Functional supply chain of hypertensive medications to ensure patients’ access to medications • Supervisory team for chronic diseases programs, including hypertension • Training of HCPs on hypertension management • Functional health information system to capture key indicators of hypertension program

**RE AIM – Program implementation**		

Adaptations to elements of EBI and implementation strategy	***Stepped-care hypertension algorithm (EBI)*** • Availability of HTN medications • Access to HTN medications • Roles of healthcare workers in the implementation of the HTN algorithm	***Team-based collaborative care (Implementation strategy)*** • Frequency of team meetings • Types of providers who joined team meetings
	***Equity considerations*** • Individual and community characteristics (e.g. poverty, rurality, language, working in the agricultural sector, and lack of formal education).	
	***Sustainability considerations*** • Having basic level of infrastructure • Transportation to visit patients in mountainous areas • Adaptations to improve feasibility and reach	***Sustainability considerations*** • Integration into primary care programs

HCPs – healthcare providers; HD – Health district; HTN – Hypertension

## Data Availability

The datasets used and/or analyzed during the current study are available on reasonable request.

## Box

**Study summary box.** Overview of implementation assessment: frameworks, selected dimensions, questions, data sources and participants

Framework / Selected dimension	Questions	Data sources	Participants

**RE-AIM / Implementation**	Program adaptations: how and why was the program adapted?	Qualitative interviews	Study staff, MoH providers and administrators
	
	Program delivery: Do implementation indicators change over time or differ between health districts?	Quantitative programmatic data	District health centers and health posts

**PRISM / Organizational perspective on the program**	What are the perspectives of health district’s providers and administrators on the program?	Qualitative interviews	Study staff. MoH providers and administrators

**PRISM / Fit between program and health district**	How did health district-level factors facilitate or hinder the program implementation?	Qualitative interviews	Study staff, MoH providers and administrators

**PRISM / Implementation and sustainability infrastructure**	What needs to be available at health districts for the program to be implemented and sustained?	Qualitative interviews	Study staff, MoH providers and administrators

**RE-AIM Extension / Sustainability**	Was the program consistently delivered over time?	Mixed methods	District health centers and health posts
	What type of program adaptations worsen or improve the program’s implementation and sustainability?		

**RE-AIM Extension / Health equity**	Was the program equitably delivered?	Mixed methods	District health centers and health posts
	What type of program adaptations reduce or promote health equity?		

## Abbreviations

BOT graph: Behavior-over-time graph
CIIPEC: INCAP Research Center for the Prevention of Chronic Diseases
cRCT: cluster randomized control trial
EBI: evidence-based intervention
HD: Health district
IECS: Institute for Clinical Effectiveness and Health Policy
INCAP: Institute of Nutrition of Central America and Panama
IRB: Institutional Review Board
LMICs: Low- and middle-income countries
MIS: Modelo Incluyente de Salud
MOH: Ministry of Health
PRISM: Pragmatic, Robust, Implementation and Sustainability Model
RE-AIM: Reach, Effectiveness, Adoption, Implementation Maintenance
RITA: Rapid Identification of Themes from Audio Recording
